# Closing the Gap: Bringing Equity to Central Line Care at Home using Quality Improvement

**DOI:** 10.1097/pq9.0000000000000835

**Published:** 2025-08-29

**Authors:** Catherine H. Murtaugh, Jean M. Abraitis, Lauren Brennan, Samantha Steich, Lindsay Brim, Lauren F. Le Goff, Lakshmi Srinivasan

**Affiliations:** From the * Department of Nursing, Children’s Hospital of Philadelphia, Philadelphia, PA; † Harm Prevention Program, Children’s Hospital of Philadelphia, Philadelphia, PA; ‡ Center for Healthcare Quality and Analytics, Children’s Hospital of Philadelphia, Philadelphia, PA; § Infection Prevention and Control, Children’s Hospital of Philadelphia, Philadelphia, PA; ¶ Department of Pediatrics, Children’s Hospital of Philadelphia, Philadelphia, PA.

## Introduction:

**Fig. 1.**
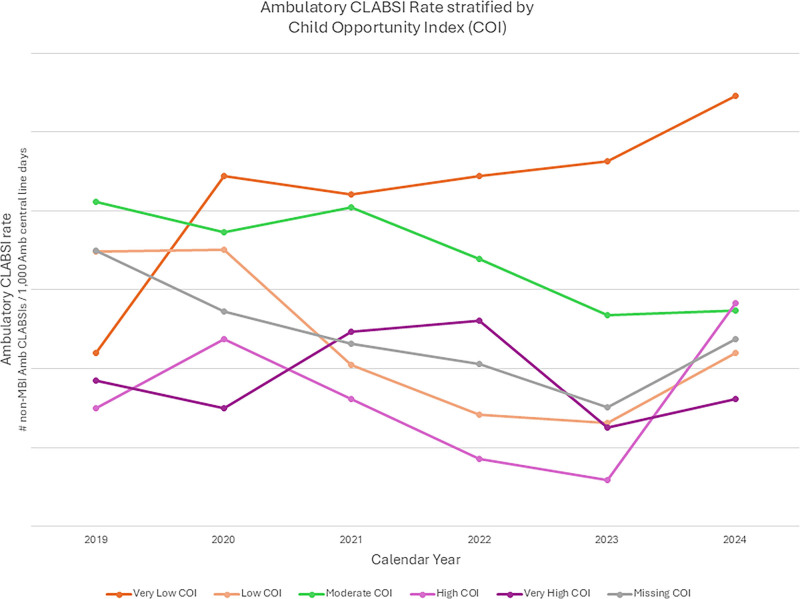
Ambulatory CLABSI rate stratified by Child Opportunity Index depicting an increased rate in the VLCOI cohort. MBI, mucosal barrier injury.

Central line–associated bloodstream infections (CLABSIs) cause considerable morbidity in children. Outpatients followed at a large, urban, tertiary care stand-alone academic pediatric hospital, living in areas classified as having “Very Low Child Opportunity Index” (VLCOI), were identified as experiencing 2-fold higher rates of non–mucosal barrier injury ambulatory CLABSI compared with other groups (Fig. 1).

We hypothesized that caregivers in VLCOI areas may experience barriers accessing infection prevention supplies, including financial strain and logistics (eg, transportation, access, time). Line care supplies (eg, dressings, flushes) are typically covered by insurance; infection prevention supplies (eg, hand sanitizer, antimicrobial wipes) are not. A global aim was set to reduce ambulatory non–mucosal barrier injury CLABSI in the VLCOI cohort by 50% by December 31, 2025, with a process aim to provide noninsurance-covered supplies to eligible patients by June 30, 2025, including all VLCOI patients.

## Methods:

**Fig. 2.**
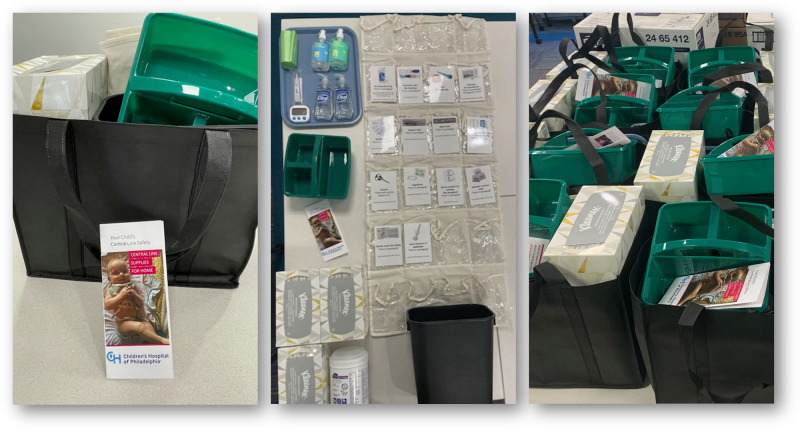
Family-Led Infection Prevention (FLIP) supply bundles offered at discharge to families caring for a central line at home.

A multidisciplinary team (inpatient and ambulatory nursing, family consultants, infection prevention, social work, home care, and patient safety) completed a fishbone and process mapping, identifying that families may have unmet supply needs. Supply bundles (Fig. 2) were designed and purchased through grant support, then assembled and distributed with information in English, Spanish, and Arabic. All families caring for patients with a central line at home were offered a bundle regardless of Child Opportunity Index status, hypothesizing that VLCOI is a proxy for general barriers to accessing supplies. Feedback was collected postdischarge.

## Results:

During 16 weeks, 52 families were approached with 39 (75%) accepting the bundle (oncology: 22/30; gastroenterology: 15/19; pediatrics: 2/3). Five VLCOI (12%) patients approached; all accepted. Feedback (n = 18) indicates the bundle is helpful in several ways: “felt supported by clinical team” (100%), “relieved financial stress” (75%), “saved time” (50%), and “felt more prepared” (50%).

Based on feedback, some supplies were increased to last the intended timeframe (2–3 mo). Additionally, 3 different bundles were created for different types of care provided at home to reduce waste. The primary bundle is an “essential” bundle with infection prevention supplies for families not providing direct line care; this is also for resupply as needed. A second bundle combines the “essential” supplies plus those for reliable care, such as a plastic tray/work surface and a timer. A third bundle adds an over-the-door organizer labeled with pictures and tips (eg, “wipe scissors with alcohol before use”); this is for families on parenteral nutrition, dialysis, or apheresis at home, as these therapies require the most supplies. A definitive assessment of the primary outcome requires additional time given sample size limitations in the stratified CLABSI data.

## Conclusions:

Supply bundles are useful and supportive for central line care at home for all families, though they may be particularly important for families living in VLCOI areas and other vulnerable groups. Achieving the global aim depends on reliable identification of VLCOI patients and wide-scale bundle distribution. Additional interventions may be required to reduce inequity and improve patient outcomes. Ongoing work aims to ensure complete capture of the VLCOI cohort and other families in need, and to establish unit- and outpatient-based systems to scale the intervention.

